# Supplementary material for: Addictive use of digital devices in young children: Associations with delay discounting, self-control and academic performance
Source: PLoS One. 2021 Jun 22;16(6):e0253058. doi: 10.1371/journal.pone.0253058 (PMC8219150; doi:10.1371/journal.pone.0253058)
Supplement: S1 Table — (DOCX) [file pone.0253058.s001.docx]

**S1 Table. Descriptive statistics for the main measures.**

|  | **Mean** | **Std. dev.** | **Min** | **Max** |
| --- | --- | --- | --- | --- |
| **Proportion of LDR [%]** | 47.4 | 26.2 | 0 | 100 |
| **DASC score** | 51.3 | 17.8 | 25 | 103 |
| **Grade average (1.0 best, 6.0 worst)** | 2.2 | 0.7 | 1.0 | 4.0 |
| **Self-control** | 45.2 | 8.7 | 23 | 65 |
