# Supplementary material for: Addictive use of digital devices in young children: Associations with delay discounting, self-control and academic performance
Source: PLoS One. 2021 Jun 22;16(6):e0253058. doi: 10.1371/journal.pone.0253058 (PMC8219150; doi:10.1371/journal.pone.0253058)
Supplement: S2 Table — (DOCX) [file pone.0253058.s002.docx]

**S2 Table***.* **Correlations between DASC subscales and key variables**

| Variable | 1. | 2. | 3. | 4. | 5. | 6. | 7. | 8. | 9. | 10. | 11. | 12. |
| --- | --- | --- | --- | --- | --- | --- | --- | --- | --- | --- | --- | --- |
| 1. Preoccupation | - |  |  |  |  |  |  |  |  |  |  |  |
| 1. Tolerance | 0.46*** | - |  |  |  |  |  |  |  |  |  |  |
| 1. Withdrawal | 0.46*** | 0.71*** | - |  |  |  |  |  |  |  |  |  |
| 1. Problems | 0.38** | 0.65*** | 0.69*** | - |  |  |  |  |  |  |  |  |
| 1. Conflict | 0.35** | 0.61*** | 0.64*** | 0.57*** | - |  |  |  |  |  |  |  |
| 1. Deception | 0.25* | 0.53*** | 0.67*** | 0.68*** | 0.53*** | - |  |  |  |  |  |  |
| 1. Displacement | 0.46*** | 0.53*** | 0.63*** | 0.71*** | 0.44*** | 0.60*** | - |  |  |  |  |  |
| 1. Relapse | 0.56*** | 0.54*** | 0.60*** | 0.62*** | 0.45*** | 0.50*** | 0.60*** | - |  |  |  |  |
| 1. Mood modification | 0.51*** | 0.62*** | 0.68*** | 0.56*** | 0.44*** | 0.48*** | 0.60*** | 0.62*** | - |  |  |  |
| 1. Self-reported usage | 0.26* | 0.23 | 0.36** | 0.41*** | 0.24* | 0.21 | 0.37** | 0.28* | 0.21 | - |  |  |
| 1. LDR proportion | -0.19 | -0.22 | -0.30* | -0.21 | -0.09 | -0.24* | -0.17 | -0.18 | -0.29* | 0.09 | - |  |
| 1. Self-control | -0.34** | -0.65*** | -0.55*** | -0.61*** | -0.40*** | -0.51*** | -0.56*** | -0.54*** | -0.62*** | -0.20 | 0.25* | - |

^*^*p* < 0.05, ^**^*p* < 0.01, *** *p* < 0.001

As an exploratory analysis, the relationships between the nine subscales of the Digital Addiction Scale and the main variables self-reported usage, delay discounting and self-control were investigated. The subscales were all intercorrelated, with the coefficients ranging from r=0.25 (Preoccupation and Deception, p=0.04) to r=0.71 (e.g. Tolerance and Withdrawal, p<0.001). Self-reported usage was positively related to the subscales Preoccupation, Withdrawal, Displacement, Relapse and Problems, the latter showing the strongest correlation of r=0.41 (p<0.001). Delay discounting, as measured via the proportion of larger, delayed reward choices, was significantly correlated with only three subscales, namely Withdrawal (r=0.30, p=0.01), Deception (r=0.24, p=0.04) and Mood Modification (r=0.29, p=0.01). Self-control was negatively associated with all nine subscales of the DASC, the strongest relationship being with Tolerance (r=-0.65, p<0.001).
