# Supplementary material for: Addictive use of digital devices in young children: Associations with delay discounting, self-control and academic performance
Source: PLoS One. 2021 Jun 22;16(6):e0253058. doi: 10.1371/journal.pone.0253058 (PMC8219150; doi:10.1371/journal.pone.0253058)
Supplement: S1 Appendix — (DOCX) [file pone.0253058.s003.docx]

**S1 Appendix. Additional correlations of self-reported screen time.**

| Variable | 1. | 2. | 3. | 4. | 5. | 6. |
| --- | --- | --- | --- | --- | --- | --- |
| 1. Self-control | - |  |  |  |  |  |
| 1. Grade average | -0.31** | - |  |  |  |  |
| 1. Self-reported usage | -0.20 | 0.43*** | - |  |  |  |
| 1. Weekday usage | -0.17 | 0.25* | 0.26* | - |  |  |
| 1. Weekend usage | -0.27* | 0.43*** | 0.50*** | 0.65*** | - |  |
| 1. Total week usage | -0.26* | 0.40*** | 0.45*** | 0.86*** | 0.95*** | - |

^*^*p* < 0.05, ^**^*p* < 0.01, *** *p* < 0.001

The table compares the measure of screen time used in the main section of this paper (variable 3; sum of daily screen times of popular digital activities) with alternative measures of screen time and their correlations with variables relevant for the investigation of hypothesis 3, namely self-control and grade average. The measure used in the main section and total week usage are moderately correlated (r=0.45, p<0.001). More importantly, the strengths of the correlations with self-control and grade average are similar (e.g. self-reported usage and grade average r=0.43, p<0.001 vs. total week usage and grade average r=0.40, p<0.001). This pattern of relationships suggests a degree of reliability of the self-reported screen time measure used in this paper and thereby increases confidence in the findings related to hypothesis 3.
