# Supplementary material for: Addictive use of digital devices in young children: Associations with delay discounting, self-control and academic performance
Source: PLoS One. 2021 Jun 22;16(6):e0253058. doi: 10.1371/journal.pone.0253058 (PMC8219150; doi:10.1371/journal.pone.0253058)
Supplement: S2 Appendix — (DOCX) [file pone.0253058.s004.docx]

**S2 Appendix. Self-control as mediator**

The pattern of correlations between the main variables suggested a potential mediating role of self-control in the relationship between addictive digital device use and delay discounting. However, as no sufficient evidence for temporal ordering of variables in the causal chain of mediation was present, the following analysis was placed in the appendix for the reader’s reference. A mediation analysis using the PROCESS macro by Hayes (2012) was performed. Based on ordinary least squares regression, this method yields path coefficients for the effects of 1) independent on dependent variable without mediator (total effect), 2) independent on dependent variable with mediator (direct effect), and 3) independent on dependent variable through the mediator (indirect effect). The tool constructed 5,000 bootstrap samples to compute 95% confidence intervals and inferential statistics. When the resulting confidence interval did not include zero, effects were deemed significant. As shown in the previous section, an effect of delay discounting on the DASC score (total effect) was observed. When the mediator was included in the model, the indirect effect (delay discounting on DASC score through self-control) turned out to be significant (ß=-0.16, CI [-0.3043, 0.0212). Furthermore, the direct effect was non-significant, implying that the relationship between delay discounting and addictive use of DDs was fully mediated by self-control. A mediation diagram is provided in Fig 1.


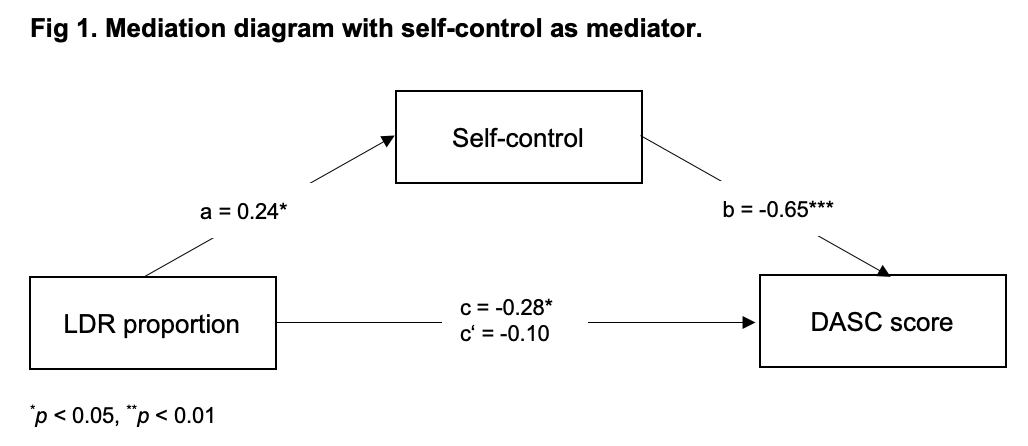


As self-control was also significantly correlated with grade average, this suggested the possibility that self-control played a further mediating role in the relationship between grade average and self-reported digital device usage. Therefore, another mediation analysis was performed, which yielded a bootstrapped confidence interval for the indirect effect that included zero, indicating that self-control was not a mediator in the relationship between self-reported usage of digital devices and the most recent grade average of participants (ß=0.04, 95 % CI [-0.0135, 0.1279]).
