# Supplementary material for: Addictive use of digital devices in young children: Associations with delay discounting, self-control and academic performance
Source: PLoS One. 2021 Jun 22;16(6):e0253058. doi: 10.1371/journal.pone.0253058 (PMC8219150; doi:10.1371/journal.pone.0253058)
Supplement: Attachment — Submitted filename: Response letter.docx [file pone.0253058.s006.docx]

Berlin, 8.4.2020

Dear Dr. Xin,

I greatly appreciate your and the reviewer’s efforts in reviewing my original submission. The reviewer’s detailed and constructive comments helped in the revision process and I am confident that I am now submitting a significantly improved paper.

Please find below my responses to each point raised by the reviewer. Note that the references to specific pages or lines refer to the Track Changes version of the manuscript.

Thank you,

Tim Schulz van Endert

**Reviewer 1**

| 1. First, I appreciate the contextualisation provided in the introduction. This helps in understanding the specific study as it is likely that the covid-19 pandemic would reasonably have had an affect on the targeted behaviour and outcomes. | I appreciate this positive comment, which requires no changes in the manuscript. |
| --- | --- |
| 2. On page 4 it is stated that: “…seems promising but is still in its infancy [11] … A central theme for the advancement of this line of research has been the development and validation of scales assessing digital addiction.” First, I am not sure what the reference to 11 adds to this particular point. Second, why is it promising, what aspect of this line of research is promising? How, after 20+ years of research on behavioural addictions (with 20+ years of research on so-called Internet addiction) is it still in its infancy. This statement should be supported. Second, I agree that a central theme in this line of research is the development of scales for assessing this construct. However, I am not convinced that this is a part of any advancement, nor am I convinced of the validation procedures that these scales have been subject to. See, for instance this paper for a satirical take on this point: https://doi.org/10.3758/s13428-020-01462-9 this paper for a qualitative investigation https://doi.org/10.1007/978-3-030-45002-1_18, this paper for an investigation of their psychometric properties https://doi.org/10.1016/j.chb.2014.09.026, and this paper for a deep dive into the genealogy of some of these scales https://www.nordicom.gu.se/sv/system/tdf/kapitel-pdf/2_lundedal-nielsen.pdf?file=1&type=node&id=39365&force= More critical engagement with the validity concerns in these measures is required. | The suggestion for improvement of this section along with provision of valuable studies by the reviewer is greatly appreciated.  Ad 1) Reference 11 is meant to indicate that “… in its infancy” is an indirect quote from the referenced paper.  Ad 2) The support for the statement is now provided in lines 69-76.  Ad 3) More critical engagement with the addiction scales is now included in lines 73-76 as well as in lines 650-652. In both cases, studies proposed by the reviewer were referenced. |
| 3. On page 5 it is noted that “Given that the discounting of future rewards is not only related to substance-based but also to behavioral addictions, this raises the question if delay discounting is also associated with addictive use of digital devices.” It is not clear which specific behavioural addictions have provided evidence for this claim. Is this drawing on the idea that pathological gambling has been linked with delay discounting in reference 18? Of the examples, this is the only reference provided that relates to a behaviour rather than substance related concern. If that is the case, and there is no other supporting evidence provided for the claim that “the discounting of future rewards is not only related to substance-based but also to behavioral addictions”, given the central importance of this argument in the position presented in the paper, the strength of this evidence is key. I note that this study includes only a sample of 62 participants. This is not automatically a problem but, it does raise questions about the statistical power. To further bolster the position that 1) delay discounting is related to maladaptive behaviours and, given this, that 2) it may also be associated with use of digital devices, more sound evidence for 1) is required. | The finding that delay discounting is related to behavioral addictions is quite robust and the references provided are just a few examples from the rich literature on the topic. To provide stronger support to the claim that delay discounting is related to problematic behavior, I added a second reference for pathological gambling and another for overeating (line 112). |
| 4. A further concern I have is with the rhetoric of addiction used throughout. Perhaps it would be better to consider concepts of compulsion, or automatic/habitual behaviour rather than problematic or pathological behaviour. See, for instance, https://doi.org/10.1007/s11097-020-09669-z | As the main theme of the paper is use of digital devices with adverse consequences (not compulsive or habitual behavior) and the major instrument used in this present study is the Digital Addiction Scale, the rhetoric of addiction – or the more tentative formulation “addictive use” used in the manuscript – seems appropriate. |
| 5. For the hypotheses, if the language of addiction is retained, addictive use of digital devices needs to be more clearly defined. | A clear definition in accordance with Hawi et al. (2019) is now provided in lines 79-85. |
| 6. Are these hypotheses pre-registered? | The hypotheses are not pre-registered. |
| 7. For H2, I am concerned about the provision of a mediation hypothesis on, seemingly, cross-sectional data. Noting that mediation represents the situation within which the effect of a third variable explains how or why two other variables related, by definition, mediation analysis implies a causal process. Despite its widespread use, examining mediation in cross-sectional data that are observational (i.e., not experimental) is generally ill advised. Examining mediation in cross-sectional data undermines an assumption of the statistical mediation model: the assumption that temporal ordering of variables in the causal chain of mediation is correct. Specifically, as many have noted (e.g., https://doi.org/10.3945/ajcn.117.152546 or https://doi.org/10.1080/00273171.2011.606718), standardized coefficients from a mediation model produced with cross-sectional data reflect a series of correlation coefficients: 1 bivariate correlation coefficient and 2 partial correlation coefficients. Any estimations of a mediation effect in such data are thus correlational in nature. Fairchild and McDaniel (2017: https://doi.org/10.3945/ajcn.117.152546) note that “the notion that correlation coefficients do not infer causation, and cannot offer insight into directionality of a relation between variables, is a lesson taught early and often in statistics classrooms.” They note further that; “Studies show that cross-sectional sample data can indicate support for mediation effects when there is no true mediation process in the population. These findings are not to say that it is impossible to examine mediation hypotheses with data that are collected at one point in time; however, there is an onus on the researcher to provide a compelling rationale that temporal ordering of the examined variables is correct.” In this case, we do not have sufficient evidence to conclude that the temporal ordering proposed is theoretically plausible. | The reviewer’s detailed and substantiated criticism of the mediation analyses is appreciated. As a consequence, the mediation analyses are now no longer part of the main text and were replaced by a simple regression analysis (pages 13-14), which seems more appropriate for the nature of the present data. Nonetheless, since many reviewers (as seen e.g. in the review process of our first paper “Likes and impulsivity: Investigating the relationship between actual smartphone use and delay discounting”) request a mediation analysis even in cross-sectional, observational studies, I decided to keep the original mediation analyses in an appendix for future reviewers’ reference.  Also, note that the abandonment of mediation analyses is further described in lines 607-608 including a reference to a key study proposed by the reviewer. |
| 8. The justification for the specific sample makeup is clear and cohesive. What is not clear is why 75 participants were selected to participate. Was this number based on a specific reasoning? See for instance this recent paper: https://psyarxiv.com/9d3yf/ | A sentence in line 194 was added that clarifies that 75 students chose to participate but some invited students (or their parents/guardians) declined to participate. |
| 9. Given recent meta-analytic evidence that indicates that these are poor reflections of actual usage, I have concerns about the use of self-report media use variables. I do note, however, that this limitation is noted by the authors in the limitations section, with reference to this recent meta-analysis. Importantly, however, this limitation also extends to beyond this single study. With particular relevance to measurement, the DASC measure is motivated as being appropriate by referencing reference 11. Notably, however, it is not clear in this paper how the scale is directly validated as a measure of addiction for digital devices (see above concerns in general) as it is merely related to self-reported usage measures (in addition to various psychometric analyses of its factor structure). See this paper for more information: https://doi.org/10.1177/2515245920952393 and this paper for further information: https://doi.org/10.1177/1948550617693063 | This criticism seems to be rather directed towards Hawi et al. (2019) and the validation of their DASC measure and less towards the current manuscript. Nonetheless, to accommodate the reviewer’s skepticism towards the validation procedure of the DASC, a limitation concerning the latter was added in lines 650-652. Studies proposed by the reviewer were referenced. |
| 10. Given the analyses conducted, the results are well-reported and, to my knowledge, conform to norms in these regard. That said, see my above comments re. Mediation in cross-sectional data. | This point requires no further changes to the manuscript. |

**References**

Hawi, N. S., Samaha, M., & Griffiths, M. D. (2019). The digital addiction scale for children: Development and validation. Cyberpsychology, Behavior, and Social Networking, 22(12), 771-778.
