# Supplementary material for: Addictive use of digital devices in young children: Associations with delay discounting, self-control and academic performance
Source: PLoS One. 2021 Jun 22;16(6):e0253058. doi: 10.1371/journal.pone.0253058 (PMC8219150; doi:10.1371/journal.pone.0253058)
Supplement: Attachment — Submitted filename: Response letter.docx [file pone.0253058.s007.docx]

Berlin, 18.5.2021

Dear Dr. Xin,

I appreciate your and the reviewer’s efforts in reviewing my manuscript. Both reviewer’s constructive comments have all been thoroughly addressed, resulting in a significantly improved paper.

Please find below my responses to each point raised by the reviewer. Note that the references to specific pages or lines refer to the Track Changes version of the manuscript. Also, all relevant data are available in the file inventory.

Thank you,

Tim Schulz van Endert

**Reviewer 1**

| 1. Regarding the data collection, given the events of the last year, to provide important context for this study, I think it is necessary for more information on the timing of data collection to be provided. i.e., did data collection occur before any lockdowns due to the pandemic, during the time of the pandemic etc.? | Details on the timing of data collection is now provided in lines 243-244. |
| --- | --- |
| 2. For the regression analyses can you please report the R^2, F-statistic, and p-value for the overall regression in the text. This applies to all three models. | The requested parameters are now included in all three models (lines 268-269, 313-314 and 531). |
| 3. It is interesting to note that in model 1 (as described in Table 2) LDR proportion is a statistically significant predictor but, in model 2 (as described in table 3), after controlling for self-control by including it in the model, LDR proportion is no longer a statistically significant predictor of DASC scores. In model 2, the only statistically significant predictor is self-control. I’m just highlighting this as a comment, as it is already pointed out on page 13. This has important implications for the discussion as, while model 1 might seem to indicate that LDR proportion and delay discounting are important predictors of addictive digital behaviours, this appears to actually have more to do with self-control than delay discounting. When the model accounts for self-control, delay discounting is no longer a statistically significant factor predicting addictive digital behaviours. While this is briefly noted in the second paragraph of the discussion, I feel that less emphasis on the finding in the first paragraph of the discussion is warranted. | I appreciate the reviewer’s suggestion to put more emphasis on the confounding role of self-control in the discussion. First, I have now elaborated on this finding and highlighted the superiority of self-control as a predictor of addictive use of digital devices in lines 729-732. Second, I have extended the discussion by speculating on a possibly causal mechanism behind the strong relationship between self-control and digital addiction in lines 740-745. |
| 4. I’m curious why model 3 (as described in table 4) did not include LDR proportion and self-control as additional control variables? | Model 3 is driven by hypothesis 3, which seeks to investigate the relationship between grade average and the DASC, the latter being replaced by self-reported usage. The control variables included in the model are standard potential confounders. LDR proportion was neither correlated with self-reported usage nor with grade average, precluding it from being a confounder in the present data. Self-control is correlated with grade average and almost significantly correlated with self-reported usage, which is why the variable is now included in model 3. Conceptually, it also makes sense to include it since it could have an influence on both self-reported usage as well as grade average. |

**Reviewer 2**

| 1. I appreciate the very nicely drawn up relationship between delay discounting and other addictive/maladaptive behaviours. The case for self-control is a bit sparse but acceptable. However the link to academic performance seems a bit out of context. I had an impression that the author intended to draw out a mediation / moderation analysis with these variables and possibly academic performance as dependent variable. I am a little surprised with the rather humble hypotheses. Isn't H1 and H2 essentially the same since they both have the same DV? | I appreciate the reviewer’s suggestion to enrich the cases for self-control and academic performance in the introduction. First, I added an explanation of the mechanism of self-control in the context of delay discounting in lines 126-128. Second, I provided some proposed reasons for the relationship between addictive behaviors and academic performance in lines 132-134. Third, I offered a strong rationale for studying the relationship between addictive use of digital devices and academic performance of young children in lines 137-140.  H1 and H2 are not the same, since delay discounting and self-control – despite being related – are different concepts, as described in the introduction. Therefore, they could have idiosyncratic relationships with addictive use of digital devices – which was confirmed by the present data. Also, I appreciate that “perceived significance” is not a criterion for publication in PLOS ONE. |
| --- | --- |
| 2. Could the author please explain why was self-reported usage excluded from the first two analyses?  From table 2, I would run the regression as (1) DV = DASC with IV; self-reported usage, LDR proportion, self-control. They are correlated and including the non-correlated ones will remove the power of an rather small sample. (2) DV = academic performance with similar IVs. | Analysis 1 (table 1) is driven by hypothesis 1, which seeks to investigate the relationship between delay discounting and the DASC. The control variables included in the model are standard potential confounders. Self-reported usage is correlated with the DASC but not with the LDR proportion, precluding it from being a confounder in the relationship between DASC and LDR proportion in the present data.  As the reviewer requested, I added self-reported usage to the second model (table 3), as it is almost significantly correlated with self-control.  Regarding model 3 (table 4), LDR proportion was neither correlated with self-reported usage nor with grade average, precluding it from being a confounder in the present data. Self-control is correlated with grade average and almost significantly correlated with self-reported usage, which is why the variable is now included in model 3. Conceptually, it also makes sense to include it since it could have an influence on both self-reported usage as well as grade average. |
| 3. Another analysis that I would like to see is the breakdown in the DASC. At times, the sub-scales provide a much more robust explanation to the predictors. For example, I would imagine that a child's propensity to have more conflict (as measured in the DASC) would give an idea to the power of the delay discounting and tolerance/withdrawal to self-control. | I thank the reviewer for the valuable suggestion to break down the DASC and investigate relationships with the main variables. The associations of the subscales with delay discounting are described in lines 259-262, with self-reported usage in lines 273-276 and with self-control in lines 299-301. Self-control indeed had the strongest relationship with the subscale Tolerance. The comprehensive correlation matrix is provided in S5 Table. |
| 4. The author could elaborate further on the second result; with delay discounting and self-control together. What possible reasons can there be to describe this in children? | I appreciate the reviewer’s suggestion to put more emphasis on the confounding role of self-control in the discussion. First, I have now elaborated on this finding and highlighted the superiority of self-control as a predictor of addictive use of digital devices in lines 729-732. Second, I have extended the discussion by speculating on a possibly causal mechanism behind the strong relationship between self-control and digital addiction in lines 740-745. |
| 5. Self-control scale did not have scoring definitions i.e. higher scores mean better self-control or not? | A scoring definition is now provided in lines 226-227. |
| 6. "A negative correlation (r=-0.28, p=0.016) between the two variables was found, implying that on average, the more often children chose the larger delayed reward, the less they addictively used digital  devices." this seems a bit convoluted and I had to re-read it a few times to understand it. I found similar writing style in other parts of results, please change them. Thanks. | The mentioned sentence was simplified in line 258. The same was done in lines 297 and 324. Any new text as part of this revision was formulated as simple as possible. |
| 7. Have the authors made all data underlying the findings in their manuscript fully available? | All relevant data are provided in the file “S3 Dataset”. |
